# Estimating Residual Life Distributions of Complex Operational Systems Using a Remaining Maintenance Free Operating Period (RMFOP)-Based Methodology

**DOI:** 10.3390/s20195504

**Published:** 2020-09-25

**Authors:** Qianyu Chen, Gemma Nicholson, Jiaqi Ye, Yihong Zhao, Clive Roberts

**Affiliations:** 1 School of Engineering, the University of Birmingham, Birmingham B15 2TT, UK; QXC762@student.bham.ac.uk (Q.C.); G.L.Nicholson@bham.ac.uk (G.N.); JXY404@student.bham.ac.uk (J.Y.); 2 Mechanical School, Yangzhou University, Yangzhou 225127, China; zhyh@yzu.edu.cn

**Keywords:** RMFOP, prognosis, remaining useful life, regression models, railway switch

## Abstract

Recent developments in the area of condition monitoring research have been targeted towards predicting machinery health condition for the purpose of preventative maintenance. Typically, published research uses data collected from rotating components (bearings, cutting tools, etc.) working in an idealized lab environment as the case study for prognosis algorithm validations. However, the operational implementation in industry is still very sporadic, mainly owing to the lack of proper data allowing sufficiently mature development of comprehensive methodologies. The prognosis methodology presented herein bridges the gap between academic research and industrial implementations by employing a novel time period for prognosis and implementing random coefficients regression models. The definition of the remaining maintenance-free operating period (RMFOP) is proposed first, which helps to transform the usefulness of the degradation data that is readily available from data short of failure. Degradation patterns are subsequently extracted from the original degradation data, before fitting into either of two regression models (linear or exponential). The system residual life distributions are then computed and updated by estimating the parameter statistics within the model. This RMFOP-based methodology is validated using real-world degradation data collected from multiple operational railway switch systems across Great Britain. The results indicate that both the linear model and the exponential model can produce residual life distributions with a sufficient prediction accuracy for this specific application. The exponential model gives better predictions, the accuracy of which also improves as more of system life percentage has elapsed. By using the RMFOP methodology, switch system health condition affected by an incipient overdriving fault is recognized and predicted.

## 1. Introduction

Prognosis and health management (PHM) is usually carried out by industrial companies to manage machine reliability that is influenced by faults or failures. In practice, prognosis information may rely mainly on the knowledge and experience of staff, which is difficult to accumulate and therefore costly [1]. Moreover, human experience is often not sufficient to accurately determine prognosis, especially for integrated systems that are influenced by complicated factors. For instance, wind turbine systems suffer from stochastic loadings due to various wind speeds day by day, which makes it challenging to determine prognosis. Considering today’s large-scale wind farms and the long distances from operation centres, the costs of manual maintenance will also be massive [2]. A similar challenge is also experienced by railway asset managers. The increasingly demanding requirements for efficient and modern asset reliability management cannot be satisfied only by expert knowledge, which in the Great Britain’s (GB) railway industry, is becoming more uncertain due to an ageing engineering workforce [1]. Meanwhile, although a significant number of generic condition monitoring models has been developed recently to automatically predict the asset health condition [1,3,4], practical implementations in industry are still very limited due to a lack of data of the required types, alongside a generic but comprehensive methodology for implementation. Collecting a sufficient amount of complete run-to-failure degradation data is quite challenging for real-world operational systems, due to the fact that operational systems are normally repaired before functional failures occur and thus not allowed to run to failure. Moreover, most research methodologies have specific mathematical assumptions and simplifications, which limits the implementation success by industry. Often, the validation data used in the prediction model is collected from an idealized lab environment, which will increase the level of risk a business has to take when trialling the particular model in reality. There is still a very limited number of generic prognosis methodologies that can estimate an operational system’s residual life distributions and validate using real-world degradation data. This study proposes a comprehensive prognosis methodology for the collection and processing of degradation data from operational systems. The industrial context is that the performance of a population of the same type of machines can provide some references but cannot accurately indicate the health condition of each individual machine, owing to the system’s complex internal physical structure and variations of external influences, such as weather challenges and usage frequency. Therefore, the stochastic properties of individual systems among the population of the same type of machines, as well as the residual life distributions of complex systems will also be investigated in the proposed methodology. Finally, an industrial case study using operational railway switch systems is used in order to assess the methodology’s effectiveness.

Strictly speaking, a system can be defined as a cluster of interrelated units that form a unified whole. A subsystem or a component is a group of elements within the system, which is capable of interacting with remaining parts of the system [5]. From the perspective of reliability, a system is subject to failure progression that may have some stochastic properties. Monitoring the behaviour via sensors that are installed on different components can give indications about the health condition of the whole system [6]. For example, components within a telecommunication system include signals, transmitters, and receivers [7]. When the signal component is heavily corrupted with noise, the transmission quality of the whole communication system will be affected. In terms of a railway switch system that diverts trains from one line to another at junctions, the main components are the points (movable rails or switch rails), guard rails (static rails), point machine (switch motor), driving rod and lock rod, reduction gear box and some bearings. The health condition of a switch system can be estimated by analyzing the sensor signals collected from its different components [8].

A prognosis is an estimation of an asset’s future health status, found by assessing current and past monitoring data; it estimates the remaining residual life before failure, which could be either a determined value or a random variable with an associated probability density function (pdf). In the determination of asset prognosis, it is currently necessary to set the assumption that the failure mode is specified. This means that the component/system is assumed to follow a specific failure progression pattern. However, it is more reasonable to abandon this assumption in reality. Alternatively, it is more meaningful to identify possible future failure modes and give the worst-case prognosis results for the affected system since an engineering asset may degrade due to any of several failure modes in real life, such as a deformed axle or a fully fractured axle. In addition to the aforementioned singular-failure-mode prognosis and multiple-failure-mode prognosis, the highest prognosis requirement is called post-action prognosis, which suggests potential actions that can retard or halt the failure progression of an affected system. Currently, machine PHM research is focused on fundamental singular failure mode analysis, which is also assumed in this paper.

Usually, condition monitoring data that are collected from installed sensors, as well as historical installation and maintenance records, are needed for prognosis. Prognosis research methods can be divided into knowledge-based methods, life expectancy methods, artificial intelligence (AI) methods, and physical methods. Knowledge-based methods estimate the residual life by comparing an observed situation with a databank of previously defined failure events. For example, an expert system is used to carry out fault prognosis of power plant energy conversion processes by formulating human expert rules in IF-THEN statements [9]. When the observed performance matches one pre-defined rule, the result will be given based on a previously encountered expert-described experience. However, the combination problem will occur when the system structure, external influences and failure causes get more complicated, because each expert rule can only describe one situation with single prediction output.

Life expectancy approaches assume that the device being monitored comes from a population of the same type of devices working under the same conditions with identical statistics. Therefore, the remaining useful life (RUL) that is given when the monitored data exceeds a pre-defined threshold is represented by a pdf. The trend evaluation method has been used to analyse the residual life distributions of a rotating bearing component, combined with Bayesian updating theories. For example, the exponential trend assumption has been applied to describe an incipient fault degradation of rotating bearings [10,11]. Later research further predicts a bearing’s residual life without involving historical degradation data [6]. An autoregressive moving average model is another common prediction approach in this category. Monitoring data from a methane compressor in a petrochemical plant was used to demonstrate the method’s effectiveness [12].

Compared with expectancy methods, AI methods can be applied when the amount of degradation data is sufficient and understanding of the physical degradation process is difficult to obtain. A multiple convolutional long short-term memory (MCLSTM) network and novel health indicator method are combined in [13] to address the remaining useful life prediction problems of rolling bearings. The results of a RUL estimation are given as the network output, with a generated mathematical expression describing the relationship between the input observations and output predictions.

Physical-based models calculate an estimated output from mathematical expressions describing component/system physical degradation behaviours. A non-linear model capturing fatigue crack dynamics was applied to compute the damage ratio with respect to time [14]. A mechanistic defect propagation model was introduced to estimate the residual life of bearings [15]. Although the degradation process can be understood easily, detailed knowledge about system behaviours at both macroscopic and microscopic levels is required for physical-based methods.

It can be observed that current PHM research is mainly focused on rotating components (bearings, cutting tools, etc.) working in an idealized lab environment. Specific research assumptions and required data volumes vary depending on the method. In practice, highly complex industrial systems, such as mining equipment, wind turbines, and railway switches, that are all exposed to external influences also experience degradation and need RUL estimations. For example, a railway switch that diverts the trains from one line to another at junctions is a complicated reciprocating system. The health condition of a switch system is influenced by internal electromechanical component deterioration and external train loadings, weather and usage frequency. By monitoring and analysing the sensor signals collected from system components, PHM for the overall railway switch system can potentially be achieved. The lack of proper fault monitoring over switch components may have catastrophic consequences, such as the Grayrigg derailment accident in 2007 [16]. Therefore, this paper targets a solution to the complex system prognosis problem by introducing a novel prognosis definition, which is based on the type of degradation data more readily available from field maintenance interventions for model training and validation. Subsequently, two well-developed expectancy regression models (i.e., linear and exponential) that are adjusted for machines with complex systems, are individually introduced to calculate and update the system residual life distributions. A comprehensive prognosis methodology is presented to describe the process of data collection and processing, as well as model selection and application. The methodology is finally validated using real-world data collected from multiple operational railway switch systems in Great Britain.

The remainder of this paper is organized as follows. Section 2 explains the process of data processing and model application within the proposed remaining maintenance free operation period (RMFOP)-based prognosis methodology. Section 3 shows the experimental and validation results for a specific railway switch system implementation. Conclusions are drawn in Section 4.

## 2. Degradation Modelling for System Signals

Failures in many industrial systems develop very slowly, over months or years. Additionally, many operational systems including railway switches are not allowed to run to failure in practice. Thus, it is difficult to collect the complete run-to-failure degradation data from the real world for the purpose of modelling analysis and result validation. Moreover, most developed prognosis methods are validated using rotating components that have been tested in an idealized and controllable experimental environment. Therefore, whether real-world complex system prognosis can be estimated using the same methods is still unknown. This section puts forward a solution to these problems by proposing a perspective on the prognosis definition, in terms of the remaining maintenance free operating period. Subsequently, the two the-state-of-the-art regression-based models to be applied: the exponential model and the linear model, are described. Because the analysis methodology and validation process for complex system prognosis are more complicated compared with component prognosis, an explicit summary of the methodology is also presented.

### 2.1. Prognosis Definition and Data Preparation

Three approaches were proposed in the literature to overcome the problem of realistic full lifecycle data shortages. In the first approach, a continuous degradation process is created artificially by fitting data collected from discrete fault severities into a smooth and continuous mathematical model [17,18,19,20]. Subsequently, some time-series processing methods including autoregressive moving average and time delay neural networks can be applied for RUL predictions. However, it is difficult to verify whether the model selections and parameter settings are correct and follow natural degradation properties because the continuous degradation data were created unnaturally without the ability to perform proper validations of its similarity to real-world data.

The second solution is to build a piece of vulnerable equipment or create bad operating environments in which to perform experiments. As such, the failure progression is faster than normal, making it easier to collect full lifecycle degradation data. A selection of thin drill-bits is considered for RUL estimations [21]. It is noteworthy that this method might be applicable for components with simple structures, such as drill-bits and bearings. Complex electromechanical systems such as wind turbines or railway switches, are less likely to find a vulnerable substitution [18].

The third approach simulates the real degradation process by establishing physical models [22]. In the case of a railway switch system, basic components such as the movable rails, the static rails and the switch motor should be modelled. The influence of train loads and the natural environment should also be considered. In comparison with the aforementioned two methods, it gives degradation waveforms that follow a simulated natural degradation. Nevertheless, it places a high demand on physical structure knowledge and specific mechanism theories. Furthermore, the modelling methods vary significantly for different machine types and fully realistic models are often difficult to generate. The lack of robustness and time-consuming nature of this approach make it less adopted in prognosis analysis.

The data problem can actually be considered from the perspective of the definition of prognosis. Most published literature argues that prognosis focuses on predicting the time at which a component/system can no longer achieve its expected function, which is normally caused by a functional failure. Prognosis is then equivalent to RUL estimation, which indicates the time gap from normal operation to functional failure. A different point in time at which to provide a prognosis is proposed here: the remaining maintenance-free operating period (RMFOP), which is defined as the residual time gap of a component/system being operated normally without maintenance intervention. The prediction outcome will therefore show the remaining suggested operating period and propose the time for the next maintenance intervention. The difficulty of obtaining enough prognosis data is also addressed since the data collected during normal operations of industrial systems, i.e., relating to operations between adjacent maintenance interventions is much easier to obtain, compared with complete life-cycle degradation data. It is noteworthy that fault prognosis instead of failure prognosis is usually considered to be superior for operational systems because many systems must not be allowed to run to failure in practice, wherever possible. A failure means the inability of an asset to do what is expected, of it, while a fault points to the potential for failure at a point when intervention to prevent the failure actually occurring may be possible [23].

The benefits of the newly proposed prognosis definition, i.e., RMFOP, compared to traditional life-cycle degradation analysis can be summarized as follows. First, it effectively addresses the problem of realistic data shortages. Data corresponding to the RMFOP can readily be collected from industrial systems without interrupting normal operations, which is of great significance in data-driven modelling analysis, such as in the life-expectancy methods and AI methods that have been introduced in Section 1. Second, compared with the traditional life-cycle analysis that predicts the time when the monitored system would experience a functional failure, the proposed RMFOP provides a more practical observation period. It focuses on the probability that a system can operate without a fault or failure until the next maintenance intervention. As such, the predicted result is a good reference for maintenance staff to optimize the maintenance intervals. The cost caused by unnecessary field visits can also be reduced. Third, it is more desirable to predict the RMFOP in situations when a functional failure would be catastrophic, e.g., train derailment or nuclear power plant accidents.

### 2.2. Model Selection

Appropriate model selection for operational systems requires an appreciation of data availability and of which model types are the best match and could achieve the desired performance under which specific assumptions. According to the literature, prognosis methods are grouped into knowledge-based methods, life expectancy methods, AI methods and physical methods. The problem of an algorithm’s complexity will occur in knowledge-based methods when the failure situation becomes more complicated, because each expert rule can only explain one failure situation with single prediction output. Physical-based methods are also not appropriate to describe complex system degradation behaviours, since it is quite challenging to construct the degradation model for a system with complex internal electromechanical components from both the macroscopic level and the microscopic level. AI methods require a huge volume of degradation data, the analysis process of which is not intuitive, potentially a barrier to adoption by industry. Instead, life expectancy methods can be clearly presented in mathematical terms. The prognosis methodologies are visualized step by step. Moreover, the calculated residual life is a random variable with derived pdfs. As such, the probability of asset failure can be easily calculated at any future time via the integral of the corresponding area below the pdf line. Therefore, considering industrial implementations and their moderate requirements for physical system knowledge and progression data, life expectancy methods may frequently be a suitable choice. Common expectancy methods include regression models, autoregressive models and hidden Markov models. Among them, regression-based models are introduced and applied in this research for two reasons. First, the process of curve fitting and regression analysis is simple. Subsequently, many regression models, such as the linear model and the exponential model that are applied in this research, can describe monotonically increasing functions, which usually represents well the incipient fault degradation process. Thus, the application of regression models matches well with the assumption of singular incipient fault prognosis stated earlier in Section 1. Regression models involve establishing parametric degradation paths (linear or non-linear) of condition monitoring data with random effects. An asset failure is considered to have occurred when the monitoring data reaches a pre-defined threshold. Therefore, a threshold is needed to implement regression methods. It is assumed that assets of the same type have the same statistical failure characteristics. The behaviour of a population of the same type of machines can provide some references but cannot precisely reflect the health progression of each individual machine, as a result of varying environments and usage patterns. Therefore, the probability distributions of stochastic parameters within the models are first estimated from a known group of degradation paths, which will be later adapted individually for each monitored asset.

#### Model 1: Linear model

The linear degradation model is usually applied when assuming that the machine degradation rate does not change dramatically with time. For instance, the wear of a brake pad is modelled by assuming the brake pad thickness decreases linearly with time. The linear degradation model is expressed as [6]:

(1) $$y\left(t_{i}\right)=c+\theta t_{i}+\varepsilon \left(t_{i}\right)$$

where $y(t_{i})$ is the monitored degradation signal at time $t_{i}$. *c* is a constant representing the initial condition of the component/system being monitored. θ is assumed to be a random coefficient following a prior distribution π(θ), which is generally unknown. It is assumed in this paper that π(θ) follows a Gaussian distribution with mean $\mu_{\theta }$ and variance $\sigma_{\theta }^{2}$. The term $\varepsilon (t_{i})$ describes the noise and transients within the signal, and is assumed to be independent and identically distributed (i.i.d.) $N(0,\sigma^{2})$. Bayesian theory then updates the distribution of θ [6]:

(2) $$p\left(\theta |y_{1},\cdots ,y_{k}\right)=p\left(y_{1},\cdots ,y_{k}|\theta \right)\pi \left(\theta \right)$$

where $y_{i}=y\left(t_{i}\right)$. $y_{i}$ signifies the degradation signal at time t(i). It gives the posterior probability of random variable θ, accounting for the observations of degradation signals from initial time $t_{1}$ to current time $t_{k}$. From Equation (2), the posterior mean ${\tilde{\mu }}_{\theta }$ and posterior variance ${\tilde{\sigma }}_{\theta }^{2}$ can be individually calculated as [6]:

(3) $${\tilde{\mu }}_{\theta }=\frac{\sigma_{\theta }^{2}\cdot sum2+\mu_{\theta }\sigma^{2}}{\sigma_{\theta }^{2}\cdot sum1+\sigma^{2}}$$

(4) $${\tilde{\sigma }}_{\theta }^{2}=\frac{\sigma_{\theta }^{2}\sigma^{2}}{\sigma_{\theta }^{2}\cdot sum1+\sigma^{2}}$$

where $sum1=\sum_{i=1}^{k}\left(t_{i}^{2}\right)$ and $sum2=\sum_{i=1}^{k}\left\{\begin{array}{c} \left(y_{i}-c\right)t_{i} \end{array}\right\}$. The probability of the residual life $T_{R}$ not being greater than time *t* is equivalent to the monitored degradation signal exceeding a pre-defined threshold *D* in a future time *t*, given the observations until current time $t_{k}$ [6]:

(5) $$\begin{array}{cc} P(T_{R}\leq t|y_{1},\cdots ,y_{k}) & =P(y\left(t+t_{k}\right)\geq D|y_{1},\cdots ,y_{k}) \end{array}$$

(6) $$\begin{array}{cc} \; & =\frac{\varphi \left(\frac{c+{\tilde{\mu }}_{\theta }t-D}{\sqrt{{\tilde{\sigma }}_{\theta }^{2}t^{2}+\sigma^{2}}}\right)-\varphi \left(\frac{c-D}{\sigma }\right)}{1-\varphi (\frac{c-D}{\sigma })} \end{array}$$

where φ(·) denotes the cumulative distribution function (cdf) of a normalized Gaussian distribution. Finally, the pdf of residual life is obtained by differentiating Equation (6) with respect to *t*. The derivation process, which is omitted here, and detailed results can be found in [6].

#### Model 2: Exponential model

The exponential degradation model is usually applied when the rate of degradation can be significantly influenced (accelerated or decelerated) by cumulative damage, such as corrosion and civil structure deterioration. The exponential degradation model is represented as [6]:

(7) $$y\left(t_{i}\right)=c\cdot exp\left(\theta t_{i}+\varepsilon \left(t_{i}\right)-\frac{\sigma^{2}}{2}\right),$$

where *c* is a constant and θ is a random variable with unknown prior distribution π(θ). Similar to the aforementioned linear model, it is assumed that π(θ) follows a Gaussian distribution with $\mu_{\theta }$ and variance $\sigma_{\theta }^{2}$. $\varepsilon (t_{i})$ is the noise term with i.i.d. $N(0,\sigma^{2})$. A logarithm is then taken on both sides of Equation (7) to simplify calculations. This yields [6]:

(8) $$x_{i}=\left(lnc-\frac{\sigma^{2}}{2}\right)+\theta t_{i}+\varepsilon \left(t_{i}\right)$$

The Bayesian updating method shown in Equation (2) is applied to the signal $x_{i}$ with logarithm amplitude. The posterior mean of θ is represented as [6]:

(9) $${\tilde{\mu }}_{\theta }=\frac{\sigma_{\theta }^{2}\cdot \sum_{i=1}^{k}\left\{\begin{array}{c} \left(y_{i}-lnc+\frac{\sigma^{2}}{2}\right)t_{i} \end{array}\right\}+\mu_{\theta }\sigma^{2}}{\sigma_{\theta }^{2}\cdot \sum_{i=1}^{k}\left(t_{i}^{2}\right)+\sigma^{2}},$$

The result of posterior variance ${\tilde{\sigma }}_{\theta }^{2}$ is the same as for the linear model. It is noteworthy that various distributions can be applied to model the failure process, such as the Gaussian, Weibull, Normal and Lognormal functions. The Gaussian distribution is assumed in the aforementioned two models due to its ability to model monotonic and gradual degradation, which matches well with the assumption of singular incipient fault prognosis stated earlier in Section 1. However, this is not always true for data collected from practical systems; the best choice depends on the system operating conditions and noise type.

### 2.3. Summary of RMFOP-Based Methodology

A summary of the steps to implement the proposed RMFOP-based methodology is presented below:

***Step 1:*** Collect *N* groups of sensor data. Each group of sensor data is a time series, recording the changes of sensor data for one specific system (machine) over a continuous operating period that has maintenance included. All sensor data is collected from the same sensor type that is sensitive to multiple failure mode progressions. The machine type for these *N* data groups, or equivalently, *N* machines should be the same.

***Step 2:*** Choose an appropriate type of characteristic pattern to extract from the sensor data. As such, among each time-series sensor data group, the original sensor data can be transformed into the characteristic patterns. The characteristic patterns are also known as degradation signals, which can directly demonstrate the machine degradation process. Examples of degradation signals are average, variance, maximum value, minimum value and slope. The problem of whether the selected degradation signals are appropriate can be considered in two directions. First, whether a maintenance threshold for the specified degradation signal has been defined by industrial standards and can be referred to. Second, whether the selected type of degradation signals can be validated in the following experiment: If the prognosis accuracy is not desirable, an alternative degradation signal might be considered.

***Step 3:*** Within each data group, extract and plot the degradation signals against the operating period. Since prognosis estimates the remaining operating period before the next maintenance intervention (the RMFOP), the complete degradation signals that are collected between two adjacent maintenance records are regarded as one realization of the fault progression from fault-free to faulty and is also called a degradation path. It is assumed that every maintenance action can effectively inspect and repair existing machine faults.

***Step 4:*** Normalize each degradation path and choose a degradation model for the normalized system signals from the linear and exponential models. It is suggested that a linear model could be applied first. Among the *N* normalized degradation paths, M(M<N) paths are used as prior information to determine the constant and the stochastic parameter prior distributions, as shown in Equation (1). Among the *M* degradation paths, fit each path with the linear expression and obtain the slope and intersection values for each path. *c* is then calculated as the average intersection value. The prior mean and the prior variance of θ are calculated as the mean and variance of the slope values, respectively. The variance of the error term $\sigma^{2}$ is estimated using a sequence of initial observations of the degradation path that has the highest fluctuation level. Normalization is achieved by subtracting the mean of the data and dividing by the standard derivation. The importance of normalization is to adjust measured values to a notionally common scale, which helps reduce the effects caused by local variables.

***Step 5:*** The prior distributions, which are calculated from the *M* paths, are regarded as the known population-wide characteristics for the set of monitored systems. For each remaining (N−M) machine, the residual life distributions are individually estimated and validated. For example, a random *i*th degradation path is chosen from the remaining (N−M) paths. When the degradation observation time ranges from initial time to for example the 10% life percentage, Equation (3) and Equation (4) are consecutively applied to update the parameter posterior distributions specifically for the *i*th system, according to the Bayesian theory. Then the cdf and pdf of residual life for the *i*th system can be calculated using Equation (5) and Equation (6).

***Step 6:*** Repeat Step 5 for the *i*th system when more observation times are available. Examples of 30%, 50%, 70% and 90% life percentages can be used. The updated residual life distributions for the *i*th system under different life percentages can therefore be obtained. The value of the life percentage is determined by the ratio of the elapsed degradation duration until the observation time to the overall degradation duration. For example, it takes 100 days for a fault-free machine to degrade to a failure state. The ratio of 10% life percentage means that by observing and collecting the machine data in the first 10 days to predict the residual life distributions. Similarly, the ratios of 30%, 50%, 70% and 90% individually represent collecting the first 30, 50, 70 and 90 days of data to predict residual life. With the increased life percentage ratios, the residual life distributions are updated with more observation data.

***Step 7:*** Repeat Step 5 and Step 6 for each remaining (N−M−1) machines. As such, the individual-specific residual life distributions have been estimated.

***Step 8:*** To check whether the linear expression is appropriate to model the degradation paths, plot the prediction errors with 95% confidence interval, considering all (N−M) validation paths at different life percentages.

***Step 9:*** Choose a different model (i.e., exponential model) and proceed through Step 4 to Step 8 as before. The difference lies in that the statistics within the linear model are obtained directly from the degradation signal amplitude, while the statistics within the exponential model are obtained from the logarithm amplitude of the degradation signals.

***Step 10:*** To validate the effectiveness of the Bayesian updating theory for both models as in Step 4 to Step 9, produce models without parameter updating in both the case of linear and exponential models. Conduct a comparison among the linear updating model, linear no updating model, exponential updating model and exponential no updating model in terms of the RMFOP prediction errors. Make a conclusion about the most suitable degradation model for the application.

The main innovations of the proposed methodology are: a novel prognosis definition that provides a more practical threshold for useful life estimations; the innovative combination and application manner with the established regression models; the superior model applicability over traditional RUL analysis because it can predict the health condition of an industrial complex system using real-world data.

## 3. Experiment and Results

To demonstrate and evaluate the RMFOP-based prognosis methodology shown in Section 2.3, the prediction accuracy is estimated using operational railway switch system degradation data that were collected in Great Britain. The data collection and processing methods cannot only be used for research purposes, it also lays a solid foundation for industrial implementations. The following section first presents the physical layout and relevant characteristics of a railway switch system, which is used to validate the proposed methodology. Subsequently, the process of data collection and processing is demonstrated for the specific implementation case. The performance of the linear model and the exponential model is also compared with conventional degradation models, which do not apply Bayesian theory for model parameter updating.

### 3.1. System Layout

**Figure 1:**
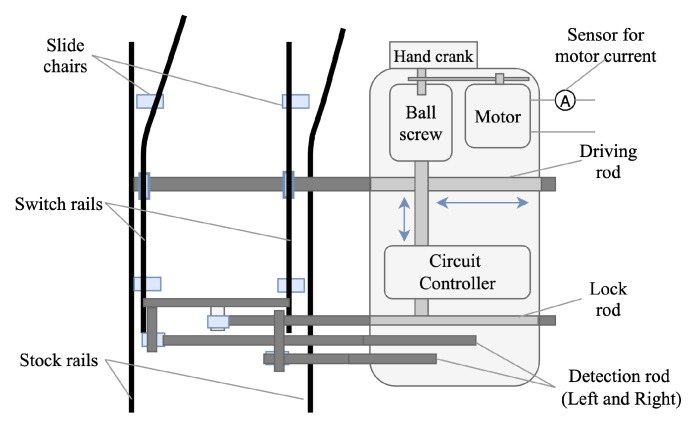
Railway switch system layout.

A railway switch, turnout, also known as a (set of) points, is a complex electromechanical system that enables trains to be diverted from one track to another. As shown in Figure 1, a switch system is composed of switch rails, stock rails, point machine, driving rod, and lock rod. The points are also known as the switch rails that can be moved laterally in either of two directions (normal or reverse). The subsystem depicted on the right is called the point machine, which slides the points from one position to another through driving rod. Railway switches can be categorised into electro-mechanical, electro-hydraulic, and electro-pneumatic types in terms of the different power operations. The electro-mechanical and electro-hydraulic-powered point machines are used more frequently. They use either mechanical transmission or hydraulic power packs actuated by electric motors as the operational power to control the mechanics. Before the motor moves the points, the lock mounted on the switches should be released. Subsequently, the motor actuates the driving rod in either direction. When completing the movement, the points will be locked in the current position, so that the passing trains can be safely diverted onto the correct track. The overall movement takes few seconds. The quality of point machine installation and maintenance has great significance in a railway performing at high capacity. This paper carries out its case study on the electro-hydraulic type of switch system.

### 3.2. Data Collection

In many applications, condition-based information can be obtained using sensor technology, the signals of which directly correlate with the physical state evolution during the degradation process. In terms of a railway switch system, the supply current and voltage of the motor can be monitored to indicate the system health condition. The tension and compression forces in the driving rod are often measured via a load pin mounted on the driving rod. The motor current sensor, as shown in Figure 1, is used in this research. Since electrical current waveforms have distinct characteristics during failure progression, which helps extract useful degradation signals for prognosis models. Additionally, current transducers are low-cost and easy to install, compared with other sensors, which reduces the difficulty for industrial implementations. By following Step 1 of the RMFOP methodology, current data were collected from fifty hydraulic switches from January 2018 to February 2019. The data obtained from each switch system is called a data group, giving a collection of fifty data groups. Specifically, for reciprocating machines such as the railway switches, each group of data is a series of event-based data records. Each record is a time series corresponding to one movement of the points.

**Figure 2:**
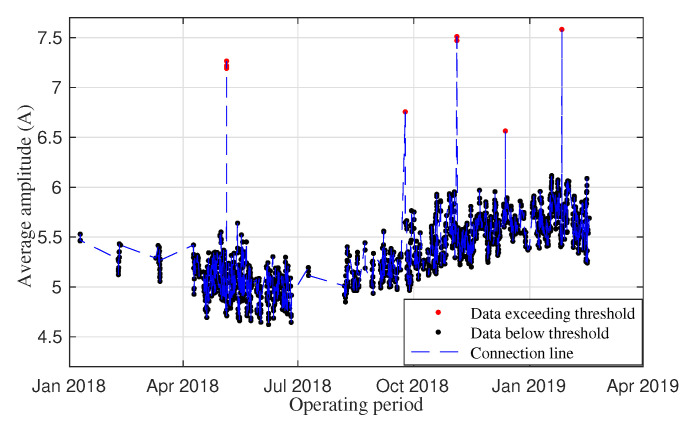
An example of degradation signals.

To find the appropriate type of degradation signal for the railway switch system in Step 2 of the RMFOP methodology, many characteristics such as variance, peak and median have been calculated and tested. The average amplitude of the time domain signal is chosen to describe the railway switch deterioration process. A maintenance threshold for average current amplitude per movement has been well defined by railway infrastructure managers. A further experiment validation process will also be conducted in Section 3.3. Another degradation signal might be used instead if the prediction accuracy found using the average amplitude is not satisfactory. The same extraction method has been previously applied in [18]. It is important to note that a well-defined threshold for the degradation signal is required to estimate the RMFOP. In the case of a railway switch system on the Great Britain mainline railway network, the maintenance threshold is defined by Network Rail, which is the infrastructure manager of most of the railway network in Great Britain. When the monitored degradation signals exceed the maintenance threshold, a fault alert is reported to the operation centre for further maintenance instructions and inspections to be carried out [24]. Following the Step 3 of the RMFOP methodology, for each switch in the dataset, the average time-domain amplitude was calculated for every switch movement and plotted against the overall operating period. Figure 2 shows the fluctuations in the average current (i.e., the degradation signal) values over the overall operating period for one railway switch. The average value of the current signal is calculated each time the point moves and are labelled as data points. The blue dashed line is drawn to link adjacent data points. The data points where the average amplitude exceeds the maintenance threshold are marked in red, while the remaining data points are marked in black. Some gaps in the data can be observed in Figure 2 between adjacent data points because the railway switch did not operate during those time periods.

**Figure 3:**
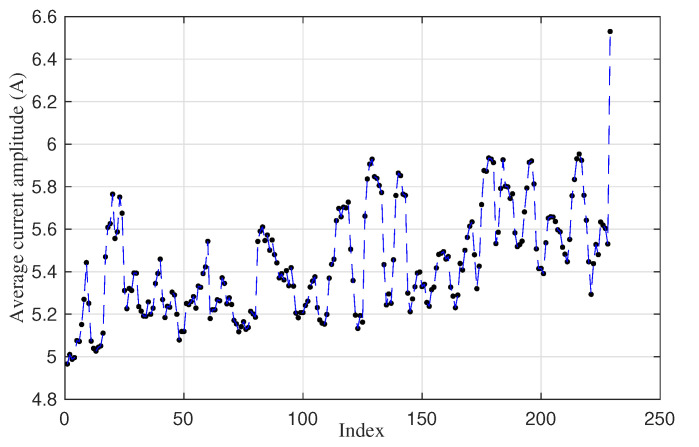
An example of degradation path.

Subsequently, the degradation signal is truncated to obtain the degradation paths. Four degradation paths with five maintenance interventions can be observed in Figure 2, when every time truncating the exceeded threshold and splitting the degradation signals into several degradation paths. It is assumed that every maintenance alert is solved properly, and the machine faults are diagnosed and fixed. Therefore, the continuous degradation signal between two adjacent maintenance records is regarded as one degradation path, which describes the continuous change of average amplitude for every point movement from fault-free to faulty state. Generally, three types of faults can influence point machine operations: abrupt faults, intermittent faults and incipient faults [25]. It is usually difficult to describe the fault progression of abrupt faults and intermittent faults. Conversely, incipient faults can be monitored and predicted when the parameters and models are appropriate in the system. In this case, only those degradation paths that show gradually increasing average values can indicate an incipient fault progression and will be retained for the following research. An example of a single degradation path is shown in Figure 3. Unlike the continuous operating time ranging from January 2018 to February 2019 in Figure 2, the switch useful life shown in Figure 3 is represented with respect to the event (a single switch movement) number, which is a time-free index. Alternatively, the system failure progression is only recorded when movement events happen. The advantage of this time-free measurement is that it does not depend on the process of the fault/failure occurrence. The example railway switch shown in Figure 3 operates approximately 230 times before launching fault alarms. Among the fifty data groups, one degradation path is generated from each data group. As such, the original fifty data groups have been processed into fifty degradation paths.

**Figure 4:**
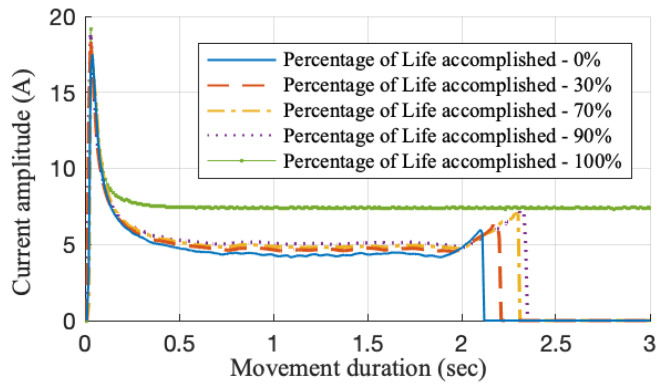
Current waveforms at different degradation percentages.

To determine the fault type, examples of the time-series current waveforms from a single point movement at different degradation percentages are shown in Figure 4. It can be observed that an incipient overdriving fault is found to be progressing. Overdriving is a fault condition where the force between the stock rail and the switch blade is beyond the ideal range (in fault-free condition) [26]. Because more electric power is needed to lock the points after moving them to the right position, an increased peak value is observed at the end of each current waveform. The movement duration is also expected to increase. When the system reaches 100% life, a continuous current supply is observed since the system overdriving failure occurs and the point can no longer be locked properly. After analysing each degradation path, it is found that the fifty paths can each represent one realization of an incipient overdriving fault progression. Among them, thirty-five degradation paths are selected at random to obtain prior model knowledge. The remaining fifteen paths are used to validate the models.

### 3.3. Results

From the two regression-based models that have been introduced in Section 2.2, the linear degradation model is applied to this railway switch system. By following Step 4 of the RMFOP methodology, the normalized thirty-five degradation paths are used as prior information to estimate the parameters in Equation (1). The constant *c* is calculated to be 0.096942. The prior mean and the prior variance of the stochastic parameter θ within the linear model are $\mu_{\theta }=0.0035892$ and $\sigma_{\theta }^{2}=$ 1.2781 × 10${}^{-5}$, respectively. The error variance estimated from the highest level of fluctuations is $\sigma^{2}=0.006$. As such, the population-wide characteristics for the monitored switch system have been obtained.

**Figure 5:**
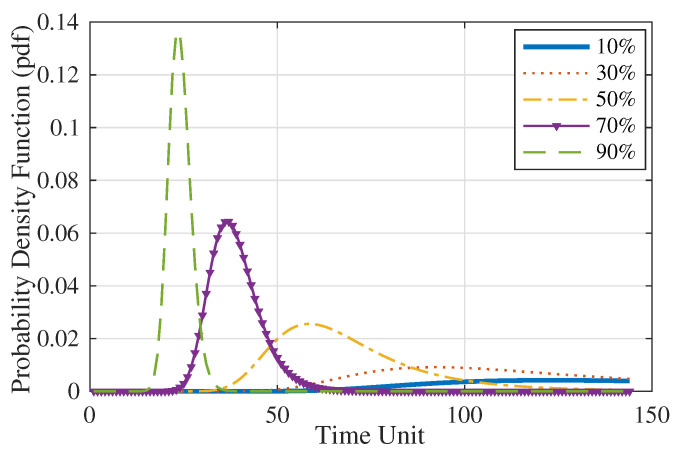
Updated RMFOP distributions of one validation switch system using the linear model.

Subsequently, the RMFOP distribution is estimated for each of the fifteen remaining validation switch systems under different life percentages, as described in Step 5 to Step 7. Figure 5 demonstrates an example of the updated residual life distributions of one validation switch system.

Step 8 is to check whether the proposed linear model is appropriate for modelling railway switch system degradation paths, the prediction errors are calculated at different life percentages for all validation paths with 95% confidence interval. The prediction error is calculated as the ratio of the predicted failure time error to the actual failure time, as shown in Equation (10). The predicted failure time is equal to the time the switch system has been operating, plus the predicted remaining life. The predicted remaining life is determined as the median of the residual life distribution because the median is a reasonable measure of the distribution central tendency. The predictions can be further improved if a close form of distribution function is derived and distribution moments are calculated.

(10) $$R_{i}^{j}=\frac{\left|\left(t_{o}^{j}+t_{p,i}^{j}\right)-t_{a,i}^{j}\right|}{t_{a,i}^{j}}$$

**Figure 6:**
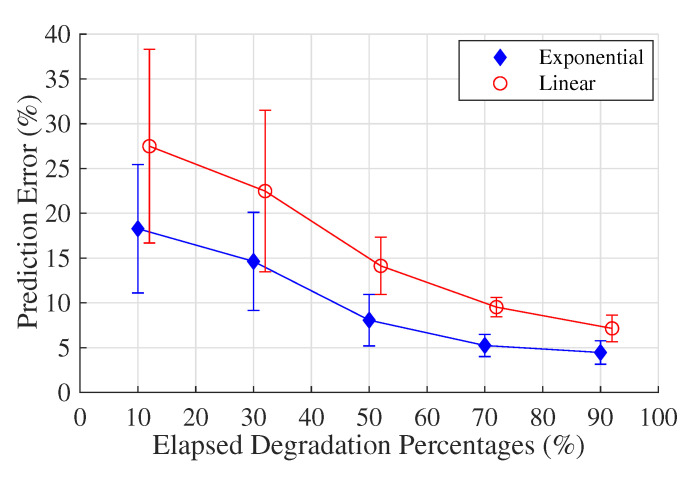
A comparison between the linear model and the exponential model regarding residual life prediction accuracy.

$t_{o}^{j}$ represents the system operating time until the *j*th observation time. $t_{p,i}^{j}$ represents the predicted remaining life obtained from the median of the *i*th system life distribution curve. $\left(t_{o}^{j}+t_{p,i}^{j}\right)$ therefore represents the predicted failure time of the *i*th system at time *j*. $t_{a,i}^{j}$ is the actual failure time and $\left|\cdot \right|$ takes the absolute error values. Hence, $R_{i}^{j}$ gives the prediction error ratio for the *i*th system at observation time *j*. In Step 9, the exponential degradation model is applied, the statistics of which are obtained from the logarithm amplitude of the degradation signals. The prediction results of the linear model are then compared with those of the exponential model, as shown in Figure 6. The linear curve is slightly offset to the right for a clear comparison.

It is clear that the prediction errors will decrease as more life has elapsed, in both the case of the linear and the exponential model. Moreover, the prediction errors are generally smaller for the exponential model, when compared with the linear model. It indicates that the exponential functional form is a better choice for characterizing the degradation process of a real-world railway switch system. This result can be attributed to the fact that the cumulative damage accelerates the switch’s natural degradation process. The results show that using the linear or exponential stochastic degradation models is a reasonable starting point to estimate residual life distributions for complex industrial applications. Therefore, considering real-world applications that have been recently retrofitted with sensors, or in which run-to-failure degradation data is difficult to obtain, the proposed RMFOP-based prognosis methodology can be applied to calculate and update the residual life distributions as more life percentage has elapsed.

**Figure 7:**
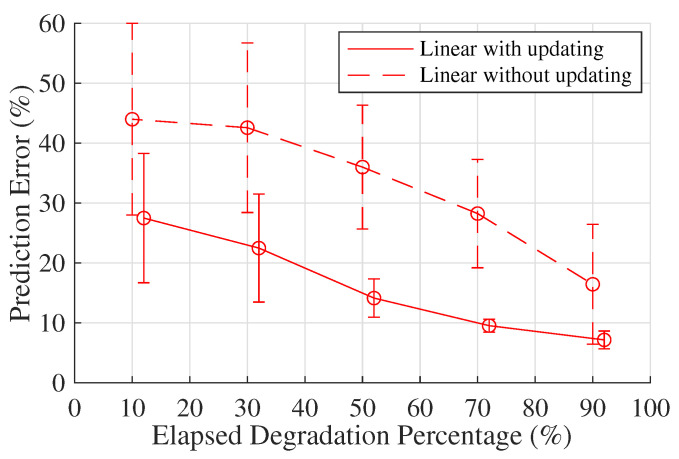
A comparison between the ‘linear with updating model’ and the ‘linear without updating model’ regarding residual life prediction accuracy.

To provide a comprehensive study of the RMFOP-based methodology shown in Section 2.3, the effectiveness of the Bayesian updating theory is validated, as described in Step 10. A comparison between the ‘linear with updating model’ and the ‘linear without updating model’ regarding the residual life prediction accuracy is drawn, the results of which are shown in Figure 7.

**Figure 8:**
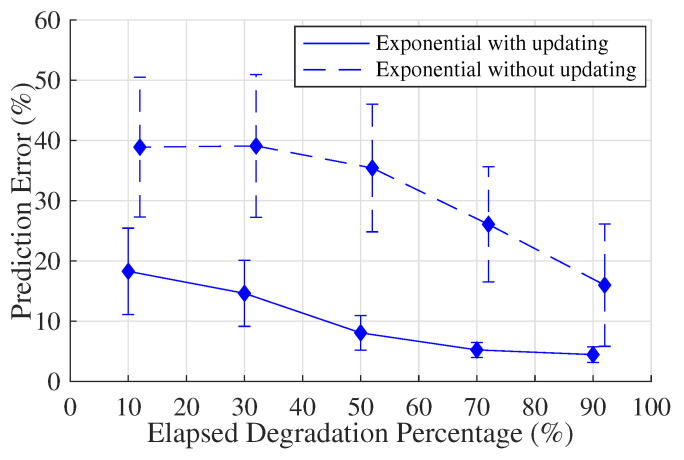
A comparison between the ‘exponential without updating model’ and the ‘exponential with updating model’ regarding residual life prediction accuracy.

It can be found that the prediction errors have been effectively decreased when applying the Bayesian theory to keep updating the parameter statistics. The same conclusion is also drawn from the comparison between the ‘exponential with updating model’ and the ‘exponential without updating model’. Figure 8 shows that the prediction errors of the ‘exponential without updating model’ fluctuate by around 30%. After updating the model parameter distributions, the prediction errors drop significantly to below 20%.

As such, a comprehensive validation of the RMFOP-based prognosis methodology has been conducted upon a railway switch system case study. The overdriving fault type makes up around 9% of Great Britain railway switch systems faults [25]. By applying this RMFOP-based prognosis methodology, the system health condition that is influenced by an incipient overdriving fault can be identified and predicted, the failure probability of which can also be calculated at any future time. However, the *K*-fold cross-validation could replace the current technique of randomly splitting data into training and validation. The *K*-fold approach consists of dividing the data into *K* partitions, training each one on *K*-1 partitions and validating on the remaining partition. The average performance of different validation results achieved in each run is a more reliable metric than random splitting.

## 4. Conclusions

This paper introduces a novel prognosis definition that focuses on the system operating period between maintenance interventions, instead of the life-cycle degradation process, the remaining maintenance free operating period (RMFOP). Therefore, the problem of appropriate degradation data shortage was addressed. Two random coefficient degradation models (i.e., linear and exponential) for calculating and updating the system residual life distributions have been introduced. Specifically, the prior distributions of the stochastic parameters are estimated based on known population-wide signals, which will then be updated individually for each validating system at different life percentages according to Bayesian theories. A comprehensive RMFOP-based prognosis methodology is provided, which discusses the steps of data collection, processing and model validation for a generic real-world operational system. The prediction accuracy is validated using real-world railway switch system data collected from Great Britain as the case study. The results indicate that both models can predict the residual life distributions for this specific industrial implementation, while the exponential model performs better in the sense that the calculated prediction errors are smaller. The errors are found to decrease when more of the RMFOP has elapsed, before dropping below 5% when more than 90% of the degradation percentage has elapsed. This suggests that the proposed RMFOP methodology paves a solid foundation for estimating residual life distributions of complex systems. As an extension in future work, a comparison between different types of characteristic pattern should be made to determine if further performance improvements are available. A more realistic degradation model could be investigated when incorporating other parameters that may also influence the prediction results, such as the operating conditions and background noise.
